# Impact of Snoring on Telomere Shortening in Adolescents with Atopic Diseases

**DOI:** 10.3390/genes12050766

**Published:** 2021-05-18

**Authors:** Keith T. S. Tung, Rosa S. Wong, Hing-Wai Tsang, Gilbert T. Chua, Dicky Chan, Kate C. Chan, Wilfred H. S. Wong, Jason C. Yam, Marco Ho, Clement C. Tham, Ian C. K. Wong, Godfrey C. F. Chan, Patrick Ip

**Affiliations:** 1 Department of Paediatrics and Adolescent Medicine, The University of Hong Kong, Hong Kong, China; keith-tung@connect.hku.hk (K.T.S.T.); rosawg@connect.hku.hk (R.S.W.); thwpaed@hku.hk (H.-W.T.); cgt560@hku.hk (G.T.C.); yutak722@hku.hk (D.C.); whswong@hku.hk (W.H.S.W.); marcoho@hku.hk (M.H.); gcfchan@hku.hk (G.C.F.C.); 2 Department of Paediatrics, The Chinese University of Hong Kong, Hong Kong, China; katechan@cuhk.edu.hk; 3 Department of Ophthalmology and Visual Sciences, The Chinese University of Hong Kong, Hong Kong, China; yamcheuksing@cuhk.edu.hk (J.C.Y.); clemtham@cuhk.edu.hk (C.C.T.); 4 Centre for Safe Medication Practice and Research, Department of Pharmacology and Pharmacy, The University of Hong Kong, Hong Kong, China; wongick@hku.hk; 5 Research Department of Practice and Policy, UCL School of Pharmacy, University College London, London WC1E 6BT, UK

**Keywords:** telomere length, adolescence, asthma, rhinitis, snoring

## Abstract

Atopic diseases can impose a significant burden on children and adolescents. Telomere length is a cellular marker of aging reflecting the impact of cumulative stress exposure on individual health. Since elevated oxidative stress and inflammation burden induced by chronic atopy and snoring may impact telomere length, this study aimed to investigate whether snoring would moderate the relationship between atopic diseases and telomere length in early adolescence. We surveyed 354 adolescents and their parents. Parents reported the adolescents’ history of atopic diseases, recent snoring history as well as other family sociodemographic characteristics. Buccal swab samples were also collected from the adolescents for telomere length determination. Independent and combined effects of atopic diseases and snoring on telomere length were examined. Among the surveyed adolescents, 174 were reported by parents to have atopic diseases (20 had asthma, 145 had allergic rhinitis, 53 had eczema, and 25 had food allergy). Shorter TL was found in participants with a history of snoring and atopic diseases (β = −0.34, *p* = 0.002) particularly for asthma (β = −0.21, *p* = 0.007) and allergic rhinitis (β = −0.22, *p* = 0.023). Our findings suggest that snoring in atopic patients has important implications for accelerated telomere shortening. Proper management of atopic symptoms at an early age is important for the alleviation of long-term health consequences at the cellular level.

## 1. Introduction 

Atopic diseases are one of the most common diseases in children and adolescents [1]. A previous study estimated that the prevalence of atopic diseases in Hong Kong could be as high as 41.2% [2], which is higher than that reported in other countries [3,4,5]. This indicates a need to estimate their burden and consequences at the population and individual levels and justify why Hong Kong provides a good context for conducting research on allergies. Although there are various types of atopic diseases with different triggers and symptoms [6], they are interrelated and share a common aetiological underpinning wherein exposure to foreign antigens may trigger a chain of inappropriate and exaggerated immune reactions [6,7]. Although the onset and course of atopic diseases vary between individuals, symptoms such as itching and swelling can be distracting and cause significant distress in patients [1]. Evidence shows that poor allergy management can negatively affect sleep quality, academic performance, psychosocial wellbeing, and health-related quality of life in children and adolescents [8]. Previous research has shown that persistent atopic diseases may accelerate the aging process and were found to be associated with other age-related diseases such as cardiovascular diseases, cancer, and even early mortality [9,10]. Telomere length (TL) also tends to be shorter in adult patients with atopic diseases [11]. 

Telomeres, known as biomarkers of aging and disease [12], are nucleoprotein complexes at the end of the chromosome that protect the genomic integrity against abnormal fusion and nucleolytic degradation. Their shortening can be attributed to genetic inheritability and environmental exposures [13]. Given that atopic patients often experience high symptom distress [14,15], increased inflammation, and oxidative stress resulting from these stressful experiences may cause chronic changes to biological pathways and are known risk factors for accelerated telomere shortening. In view of their effects on TL, the onset time and progression of allergic symptoms are believed to have important implications for long-term health [16]. 

In addition to atopic symptoms, quality of sleep is often compromised in atopic patients. Notably, snoring occurs when air is forced through the partially collapsed oropharynx or hypopharynx during sleep [17]. Previous studies have highlighted an association between snoring and atopic diseases such as asthma [18]. Snoring is also associated with an increased risk for cardiovascular diseases, high blood pressure, metabolic syndrome, and dyslipidemia [19,20]. The presence of snoring may also affect cellular functions. For example, a recent study found shorter telomeres in patients with snoring when compared to healthy individuals [21]. However, little has been done to clarify the effect of co-occurrence of snoring and atopic symptoms on cellular functions, particularly, in young populations. There is also limited research as to whether snoring represents a sign of poor symptom control in atopic patients. The notion of accelerated telomere shortening in children with co-occurrence of snoring and atopic symptoms is possible but requires research to substantiate. Therefore, the present study investigated whether atopic diseases and snoring were independently and significantly associated with TL in Chinese adolescents. It also assessed the impact of snoring on TL among adolescents with and without atopic diseases.

## 2. Methods

### Study Design and Participants

This study included a subset of participants from the HealthyKids cohort study, which was formulated in 2011–2012 to examine the long-term impact of early-life socioeconomic status on health and development. All the participants were recruited from randomly selected kindergartens in Hong Kong when they were 5 years of age. To be eligible for participation in the HealthyKids cohort study, parents had to be able to read Chinese and their children had to be in the final year (K3) of the selected kindergartens at the time of recruitment. Further details of the longitudinal cohort study can be found in previous publications [22,23]. To increase the cohort size, chain-referral sampling was adopted in each follow-up wave (2014–2015 for wave 2, and 2018–2019 for wave 3). Specifically, families in the HealthyKids cohort were approached and invited to complete a comprehensive set of questionnaires. Invitations were also sent to other eligible families in the same schools where the cohort children were attending. The present study used the data collected in wave 3 when the students reached grades 7–8. Upon obtaining informed consent, parents completed questionnaires on their family demographics and smoking behavior as well as their adolescent children’s history of atopic diseases and frequency of snoring. Buccal swab samples were also collected from the adolescents. Those adolescents who did not provide buccal swabs were not included in this study. 

## 3. Measures

### 3.1. Adolescents’ Telomere Length 

Genomic DNA was isolated and extracted from collected buccal swab samples using the QIAamp DNA Mini kit (Qiagen, Hilden, Germany) to determine the TL according to the procedures described in previous studies [24]. The extracted genomic DNA was handled in triplicate to determine the average TL using quantitative polymerase chain reaction. The TL was expressed as a relative ratio of the telomere repeat copy number (T) to single-copy gene 36B4 copy number (S) using the formula of T/S = 2^−ΔCt^, where ΔCt is the mean difference between the threshold cycle (Ct) value of the 36B4 gene and telomere repeats. 

### 3.2. Adolescents’ History of Atopic Diseases and Presence of Snoring in the Past 4 Weeks 

Parents were asked whether their adolescent children had been diagnosed with any type of atopic diseases including asthma, allergic rhinitis, eczema, and food allergy due to a prior occurrence of severe allergic reactions and whether they snored during sleep in the past 4 weeks. 

### 3.3. Demographics Characteristics 

Child age and gender were self-reported by the adolescents on the date of buccal swab sample collection. On the other hand, parent-reported family demographic information included parental marital status, family monthly income, the status of Comprehensive Social Security Assistance (CSSA), and paternal and maternal education level and smoking behavior. 

### 3.4. Data Analysis 

All analyses were conducted using SPSS Statistics (version 26.0). Descriptive statistics were used to summarize adolescents’ demographic and family characteristics. A series of independent *t*-tests (for continuous variables) and chi-square analyses (for categorical variables) were conducted to detect differences in sociodemographic characteristics and telomere length between adolescents with and without atopic diseases. To examine the risk of snoring among adolescents with atopic diseases overall and by specific disease type, a series of logistic regression analyses were adopted. Linear regression analyses were conducted to examine the independent associations of telomere length with snoring and atopic diseases. Subsequently, the models were further controlled for other sociodemographic confounders (adolescent’s age and gender and parent’s smoking behavior at home and family income level). The impact of co-occurrence of snoring and atopic diseases on TL was tested using hierarchical linear regression analyses. Interaction terms were created and included in the regression model with snoring and atopic disease history as independent variables using a block entry method. With previous evidence suggesting the associations of obesity with snoring and TL, the association between body weight status and snoring was examined using linear regression analysis. All tests were two-tailed with *p* < 0.05 denoting statistical significance. 

### 3.5. Ethical Approval

The study protocol was approved by the Institutional Review Board of the University of Hong Kong/Hospital Authority Hong Kong West Cluster (UW 18-057) on 1 March 2018. Written informed consent was obtained from all participants in this study. 

## 4. Results

A total of 354 participants were included in our analyses. Table 1 shows the sociodemographic characteristics of the participants overall and by a history of atopic diseases. The overall sample had 130 boys (36.7%) and 224 girls (63.3%) with an average age of 13.3 years. Their average monthly household income was HKD 53,049 (USD 6801). Nearly 85% of the parents were married, and over 30% of the mothers and fathers attained tertiary education or above. About 20% of the adolescents had snoring in the past 4 weeks. Approximately half of the adolescents had a diagnosis of atopic disease as reported by their parents. Specifically, 20 had asthma, 145 had allergic rhinitis, 53 had eczema, and 25 had a food allergy. The risk of atopic disease was higher in boys and those with less-educated mothers. Compared to adolescents without atopic diseases, those with atopic diseases were more likely to snore in the past 4 weeks. 

**Figure 1:**
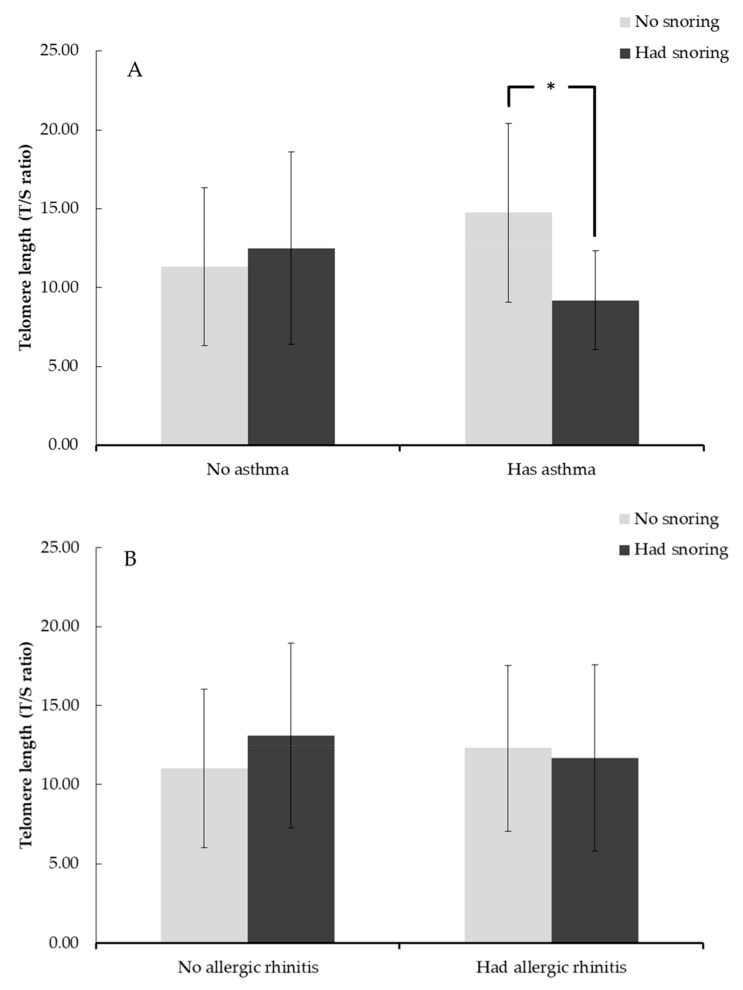

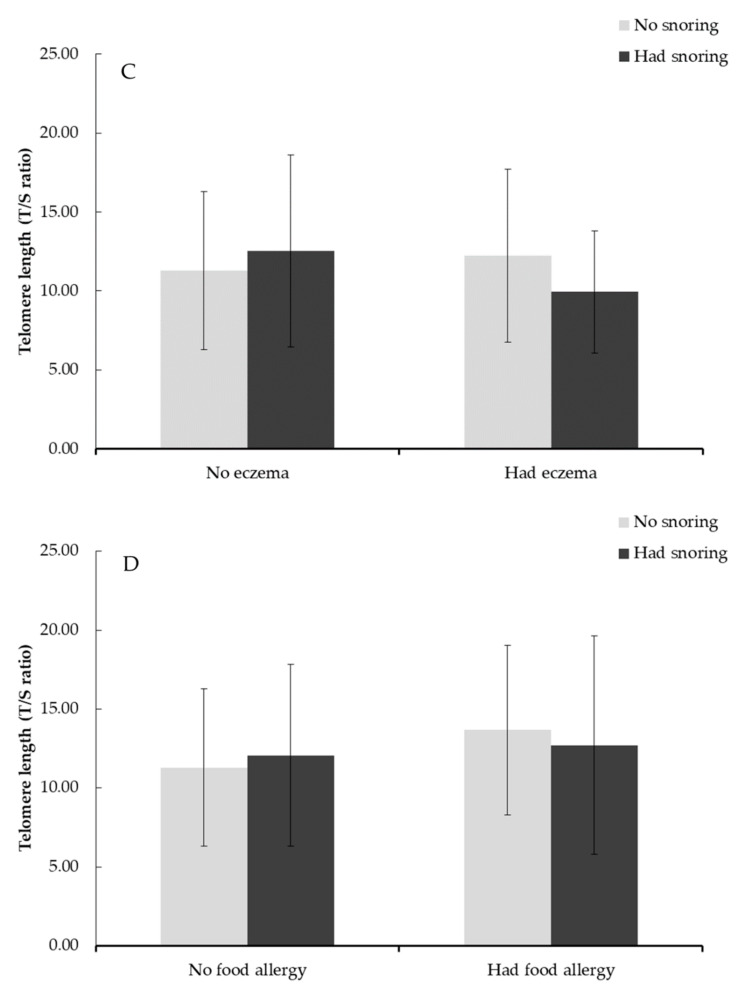

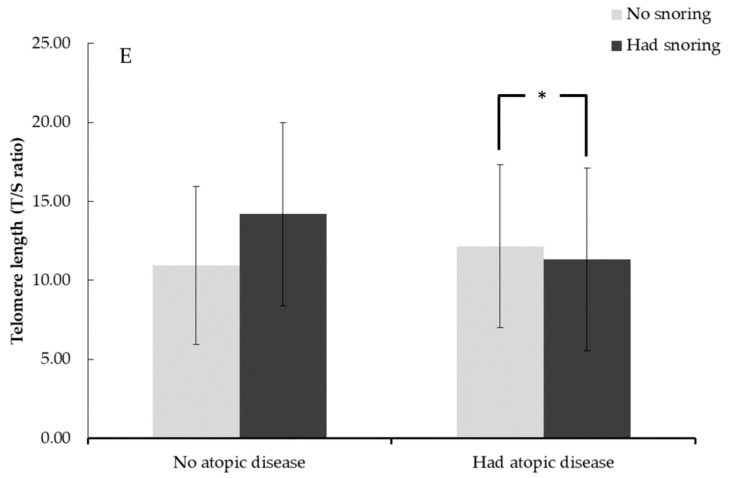
Telomere length of adolescents with or without snoring by a parent-reported history of (**A**) asthma, (**B**) allergic rhinitis, (**C**) eczema, (**D**) food allergy, (**E**) any atopic diseases. Note: * *p* < 0.05.

Compared to those without atopic diseases, adolescents with atopic diseases were more likely to snore in the past 4 weeks (aOR = 2.82, *p* < 0.001) and have asthma (aOR = 3.28, *p* = 0.001) or allergic rhinitis (aOR = 3.08, *p* < 0.001). There were no significant differences in the likelihood of snoring in the past 4 weeks between adolescents with a food allergy or eczema and those without such diseases. As shown in Table 2, neither atopic disease history nor snoring status was independently associated with adolescent TL. However, the effects on TL were significant when history of snoring and atopic diseases co-existed (β = −0.34, *p* = 0.002), particularly for asthma (β = −0.21, *p* = 0.007) and allergic rhinitis (β = −0.22, *p* = 0.023) (Table 3). Further, our regression analysis on the association between snoring and body weight status found that there is no significant association observed. Figure 1a–e illustrates the average TL by snoring status and history of atopic disease. The effect of snoring on adolescent TL was particularly strong and significant in the atopic disease group (β = −0.19, *p* < 0.05) and the asthma group (β = −0.59, *p* < 0.05). 

## 5. Discussion

This study assessed the independent and combined effects of atopic disease and snoring on adolescent TL. This is an important research question because adult TL is, by and large, determined by telomere dynamics in childhood [25], and stressors in childhood, compared to stressors in adulthood, may have stronger influences on TL. In the present study, we found that the presence of either atopic disease or snoring alone had no association with adolescent TL. However, the co-occurrence of atopic disease and snoring was significantly associated with shorter TL in early adolescence. 

This study contributes to the literature on atopic disease and aging by demonstrating the effect of the interplay between snoring and atopic disease on adolescent TL. Our findings suggest that the presence of snoring in young atopic patients could be a sign of poor symptom control and indicate a potential of progression to more severe conditions. Previous studies have demonstrated that persistently high stress can result in sizable telomere attrition [26]. As allergies often start in childhood and early adolescence [27], chronic symptom burden may cause lifelong stress that gradually takes a toll on physical and mental health and may even leave a mark on the patient’s DNA [12,18,28]. Consistent with this finding, we observed shorter TL among adolescents with the dual burden of atopic disease and snoring than those with either snoring or atopic disease. A possible explanation for this finding is the elevation of inflammation and oxidative stress caused by the stacking burden of atopic disease and snoring [11,21]. For example, the mechanical stress that arises from repetitive vibrations and airway pressure gradients during snoring may stimulate systemic inflammatory responses [29,30]. Previous research has also reported that sleep problems tend to be more severe in patients with intermittent or persistent atopic conditions [18]. Snoring may accompany intermittent hypoxia, which can increase oxidative stress due to the production of reactive oxygen species (ROS) [31]. Furthermore, atopic diseases are generally characterized by chronic activation of the immune system which has been found to trigger inflammation and oxidative stress [6,7]. Because of the high level of ROS, this pro-oxidant environment has been suggested by previous studies as a key factor contributing to accelerated telomere shortening [32,33]. Hence, early adoption of proper allergy management strategies would be important to mitigate the negative health consequences at the cellular level. 

Consistent with previous findings [34], we observed a higher likelihood of snoring among adolescents with asthma or allergic rhinitis perhaps because of the unique physiological mechanisms underlying these two diseases [34,35]. Specifically, the inflamed and narrowed upper airway associated with allergic rhinitis may cause snoring due to the increased level of upper airway resistance [35,36]. On the other hand, asthma is a lower airway problem that can cause snoring through impaired respiratory control. Apart from these physiological explanations, shared symptoms such as airway inflammation due to allergic rhinitis or asthma may provoke the release of inflammatory molecules such as cysteinyl leukotrienes which play an important role in the development of snoring [37]. 

Based on further subgroup analyses by atopic disease type, we found that the cellular impact of co-occurrence of atopic diseases and snoring was particularly strong in adolescents with asthma or allergic rhinitis. Recent studies proposed an integrated view of asthma and allergic rhinitis as “united airway disease”. It was suggested that asthma and allergic rhinitis represent the manifestations of one syndrome affecting two different parts of the respiratory tract. Both asthma and allergic rhinitis involve the process by which the inflamed airway becomes hyper-responsive toward airborne allergens [38]. Although the location of hyper-responsiveness differs between asthma (at the lower airways) and allergic rhinitis (at the upper airways), they share similar pathologic features in terms of the profile of inflammation, mediators, and adhesion molecules [35]. For example, both diseases involve IgE-mediated allergic reactions that can trigger airway inflammation due to increased TH2-type immune responses [39]. Various cells such as airway epithelial, fibroblasts, smooth muscle cells, and macrophages would release eotaxin-1 that causes eosinophil recruitment and activation [40]. The release of ROS from eosinophil activation by the TH2 cells contributes to the oxidative stress load that can incite airway inflammation [41]. A positive feedback loop of oxidative stress, inflammation-induced cell recruitment, and stem cell differentiation is thus created and might cause irreversible cellular damage such as tissue dysfunction and a high frequency of cell senescence. However, such a positive feedback loop would not be possible in adolescents with a food allergy and eczema, as their hyper-responsiveness and inflammatory process occur in different locations. It has been reported that the immune reactions triggered by food allergy and eczema mainly occur in the gastrointestinal tract and skin [42,43]. Our finding that food allergy or eczema did not interact with snoring to affect TL in adolescents is therefore in line with the existing knowledge concerning the association of snoring with oxidative and inflammation stress in the airway.

Nevertheless, this study found no significant changes in TL when the effects of atopic disease and snoring were mutually adjusted, perhaps because the burden resulting from atopic disease or snoring alone may not be large enough to trigger cellular damages in early adolescence. Indeed, previous studies proposed several mechanisms that might counteract oxidative damages to telomere DNA under mild oxidative stress exposure [44,45]. For example, basic lesions due to oxidative stress could promote the action of telomerase by inhibiting the binding of proteins to telomeres. The activation of telomerase can in turn enhance the telomerase-dependent telomere repeat additions, which may result in the lengthening of telomere [46]. Alternatively, the presence of antioxidants might also attenuate the oxidative damage to telomere DNA under mild oxidative stress [45]. However, with the co-occurrence of snoring and atopic disease, high levels of oxidative and inflammatory stress may activate a chain of DNA damage responses that contribute to the complete uncapping of telomere DNA [44,47]. Our findings, together with a growing body of evidence on the link between telomere shortening and chronic diseases, suggest that the chronic conditions of untreated atopic reactions and sleep problems may result in irreversible cellular damage that ultimately contributes to comorbidities and potentially premature mortality later in life.

However, the findings of this study should be interpreted with the following caveats: First, this was a cross-sectional study and therefore we were not able to establish a causal relationship between atopic disease and TL. Second, telomere length in this study was determined using buccal swab DNA instead of DNA extracted from peripheral blood. Despite potential differences in TL between cell types, a previous study has demonstrated a high correlation between estimates of TL determined from different samples (buccal cells, fibroblasts, and blood cells) [48]. Moreover, the validity of the use of buccal DNA in TL determination has been scientifically proven [12]. Third, we relied on parent-proxy reports to collect data on the adolescents’ history of atopic disease and the presence of snoring, which could be subject to recall bias. Data from electronic health records should be included in future studies when assessing the impact of atopic diseases. Lastly, we did not collect data on the time of onset and severity of atopic diseases. Given that the long-term health consequences may vary between childhood-onset and adolescence-onset atopic diseases [11], future research examining predictors of changes in TL should record the onset time of allergies. Furthermore, a detailed assessment of allergic symptom profiles will further help clarify the impact of allergies on telomere shortening. 

Despite these limitations, the results of this study highlight the importance of proper management of atopic symptoms during childhood and adolescence. Our findings contribute to the current evidence base by demonstrating the impact of the interplay between snoring and atopic disease on adolescent TL. The findings also highlight the potential role of snoring as a useful marker of symptom severity for atopic patients. It would be helpful to conduct regular sleep assessments in allergy clinics for early symptom detection and treatment. Finally, although atopic diseases are mainly regarded as childhood diseases, emerging evidence documents an association between atopic diseases and age-related diseases. Future studies should examine how the time of onset and trajectory of atopic symptoms during childhood and adolescence affect the aging process. Further studies are also needed to elucidate the mechanism underlying the development of comorbid atopic diseases and related chronic conditions.

## Figures and Tables

**Table 1:** Demographics of the participants.

	All (*n* = 354)	Any Atopic Diseases (*n* = 174)	Without Atopic Diseases (*n* = 180)	
	N (%)/Mean (SD)	N (%)/Mean (SD)	N (%)/Mean (SD)	*p*
Adolescent’s characteristics				
Sex				<0.001
Boys	130 (36.7%)	126 (72.4%)	98 (54.4%)	
Girls	224 (63.3%)	48 (27.6%)	82 (45.6%)	
Age	13.3 (0.6)	13.3 (0.6)	13.3 (0.5)	0.777
Parent-reported history of snoring				<0.001
Yes	74 (20.9%)	55 (31.6%)	19 (10.6%)	
No	276 (77.9%)	116 (66.7%)	160 (88.9%)	
Parent-reported history of atopic diseases	174 (49.2%)	-	-	
Asthma	20 (5.6%)	-	-	
Allergic rhinitis	145 (41.0%)	-	-	
Eczema	53 (15.0%)	-	-	
Food allergy	25 (7.1%)	-	-	
Adolescent’s Telomere length (T/S ratio)	11.6 (5.3)	12.0 (5.4)	11.3 (5.2)	0.205
				
Family characteristics				
Current marital status				0.449
Single/divorced	40 (11.3%)	19 (10.9%)	21 (11.7%)	
Married	300 (84.7%)	147 (84.5%)	153 (85.0%)	
Family monthly income (USD)	6801.2 (4724.7)	7218.2 (4590.2)	6396.1 (4830.4)	0.105
Parent smoking at home	28 (7.9%)	12 (6.9%)	16 (8.9%)	0.425

**Table 2:** Association of history of atopic diseases and snoring with adolescent telomere length.

Regression Model	Factors	Univariable		Bivariable ^a^		Adjusted for Confounders ^b^	
		β (95%CI)	*p*	β (95%CI)	*p*	β (95%CI)	*p*
1	Atopic diseases	0.06 (−0.05, 0.16)	0.270	0.05 (−0.06, 0.16)	0.338	0.05 (−0.06, 0.16)	0.400
	Snoring	0.03 (−0.07, 0.14)	0.547	0.02 (−0.09, 0.13)	0.693	0.01 (−0.10, 0.12)	0.848
2	Asthma	0.02 (−0.08, 0.13)	0.653	0.02 (−0.09, 0.13)	0.734	0.02 (−0.09, 0.13)	0.751
	Snoring	0.03 (−0.07, 0.14)	0.547	0.03 (−0.08, 0.14)	0.556	0.02 (−0.09, 0.13)	0.732
3	Allergic rhinitis	0.08 (−0.02, 0.19)	0.125	0.08 (−0.03, 0.19)	0.175	0.07 (−0.04, 0.18)	0.205
	Snoring	0.03 (−0.07, 0.14)	0.547	0.02 (−0.09, 0.13)	0.672	0.01 (−0.10, 0.12)	0.828
4	Eczema	0.004 (−0.10, 0.11)	0.940	0.004 (−0.10, 0.11)	0.944	−0.0005 (−0.11, 0.11)	0.993
	Snoring	0.03 (−0.07, 0.14)	0.547	0.05 (−0.06, 0.15)	0.390	0.03 (−0.08, 0.14)	0.560
5	Food allergy	0.08 (−0.03, 0.18)	0.150	0.07 (−0.03, 0.18)	0.169	0.07 (−0.04, 0.18)	0.201
	Snoring	0.03 (−0.07, 0.14)	0.547	0.04 (−0.07, 0.14)	0.476	0.03 (−0.08, 0.13)	0.630

^a^ In the bivariable, we adjusted for history of atopic diseases and snoring status to test independent associations. ^b^ Multivariable model with (a) further adjusted for adolescent’s age and sex, parent’s smoking behavior at home, and family income level.

**Table 3:** Association of co-occurrence of atopic diseases and snoring with adolescent’s telomere length.

	Adolescent Telomere Length	
	β (95%CI)	*p*
Atopic disease × Snoring	−0.34 (−0.54, −0.13)	0.002
Asthma × Snoring	−0.21 (−0.37, −0.06)	0.007
Allergic rhinitis × Snoring	−0.22 (−0.41, −0.03)	0.023
Eczema × Snoring	−0.11 (−0.23, 0.02)	0.105
Food allergy × Snoring	−0.06 (−0.19, 0.08)	0.407
Adjusted for adolescent’s age and sex, parent’s smoking behavior at home, family income level, and the main effect of corresponding atopic disease and snoring		

## Data Availability

The data that support the findings of this study are available from the corresponding author upon reasonable request.
